# Intrauterine Device Migration: A Diagnostic and Management Dilemma

**DOI:** 10.7759/cureus.57637

**Published:** 2024-04-05

**Authors:** Sneha Venkataramani, Mohamed M Elkott, Pamela O Restrepo, Samah K Elkhider, Jalal Alshareef, Sadoon S Sadoon

**Affiliations:** 1 Department of Obstetrics and Gynaecology, NMC Royal Hospital, Khalifa City, Abu Dhabi, ARE

**Keywords:** copper iud, laparoscopy, intrauterine device migration, irregular menstruation, abdominal pain

## Abstract

Intrauterine devices (IUDs) are an effective method of contraception, with failure rates comparable to sterilization. In rare cases, IUDs can migrate to other sites, including the bladder, cecum, and fallopian tubes. This case reports a 44-year-old woman who was misdiagnosed with a urachal cyst due to the migration of her IUD into the anterior abdominal wall. A laparoscopic retrieval was successfully performed. To prevent any further serious complications, it is imperative to promptly diagnose and manage migrated IUDs.

## Introduction

Among the most effective forms of contraception available, intrauterine devices (IUDs) have failure rates similar to those of sterilization [1]. At present, copper-containing IUDs and levonorgestrel-containing IUDs have similar success rates in preventing pregnancy, with failure rates of 0.08% and 0.02%, respectively [1]. However, there are a few complications with the insertion of IUDs. The most frequently noted side effects include pelvic pain and heavy menstrual bleeding. Rare complications include pregnancy, displacement and migration of the IUD, and uterine perforation [2,3]. Data suggest that IUD migration is rare with less than one case reported for every 500 patients [4]. The migration of IUD is often noted to sites such as the urinary bladder, cecum, and pouch of Douglas via the uterus and the fallopian tube [4,5]. IUD migration can present with a variety of abdominal symptoms and signs based on the site and severity of involvement [5]. Radiological investigations might help diagnose the lost IUD. If the radiological investigations are inconclusive, further workup for the patients would include hysteroscopy and diagnostic laparoscopy [5]. Immediate retrieval of the IUD by diagnostic laparoscopy is important to prevent further complications.

We are presenting a case of IUD migration which was initially diagnosed as a urachal cyst on MRI. It highlights the challenges in the diagnosis and management of such conditions.

## Case presentation

A 44-year-old female gravida 4 para 4 presented to the outpatient clinic with mild lower abdominal pain and irregular menstruation for the last few months. Obstetric history revealed four previous pregnancies delivered by normal vaginal delivery and cesarean section. No complications were noted during the pregnancies. Eight weeks post her third pregnancy, she had a copper IUD inserted. However, during a follow-up, the IUD could not be located despite all investigations. Eventually, she became pregnant, and her baby was delivered by cesarean section. The patient had an Implanon for two years and was removed recently. Gynecological history revealed irregular menstruation. She was a known case of uterine leiomyoma. No consanguinity was noted. Family history was significant for type 2 diabetes mellitus and hypertension.

**Figure 1:**
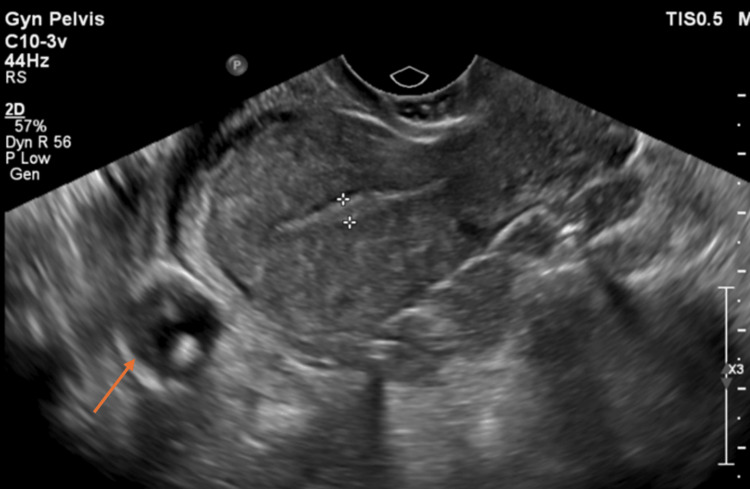
Transvaginal ultrasound showing an anterior abdominal wall cyst.

**Figure 2:**
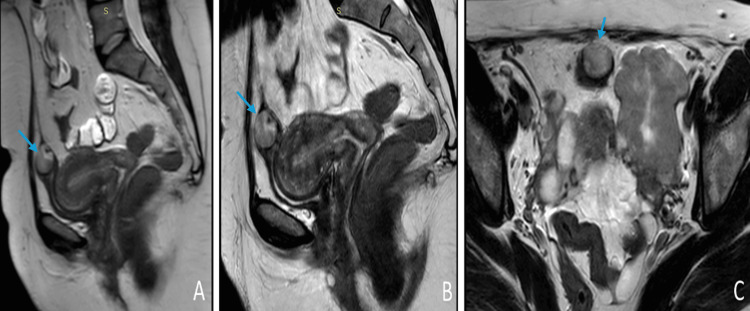
MRI of the pelvis. A, B, and C denote the presence of a cystic mass (urachal cyst) in the anterior abdominal wall (as indicated by arrows).

A transvaginal ultrasound (Figure 1) reported a well-defined, non-homogenous, hypoechoic, left adnexal solid lesion with multiple intervening echogenic hyperreflective as well as lucent cystic foci measuring 4.8 × 4.9 × 5.2 cm. The adnexal lesion was seen at the interface between the uterus and the left ovary (the latter was within the normal size with a dominant follicle measuring 9 mm). The right ovary appeared normal in size and morphology showing dominant anechoic follicular cyst. No cystic or solid lesions were noted. A further workup with pelvic MRI (Figure 2) revealed a left adnexal lesion with imaging characteristics of a fibroid- suggesting a broad ligament fibroid. The urinary bladder was partially distended. A well-defined, rounded, non-enhancing cystic lesion measuring 2.3 × 2.2 cm was seen, arising from the dome of the urinary bladder and mimicking a urachal cyst.

**Figure 3:**
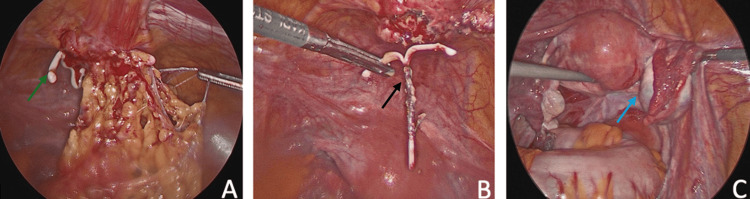
Laparoscopic findings. A: Cystic collection with the migrated IUD (green arrow). B: Retrieved IUD (black arrow). C: Ovarian mass (blue arrow). IUD: intrauterine device

In view of her findings, the patient underwent laparoscopic mass excision of the left adnexal mass. Laparoscopic findings (Figure 3) included omental adhesions to the anterior midline abdominal wall and a left adnexal mass measuring about 7 cm. A copper IUD surrounded by a collection which presented as a mass found in the anterior abdominal wall was drained. Swabs were obtained for culture, and it was negative. The surgery was uncomplicated, and she had an uneventful hospital course. Pain was well-controlled on oral medications, and she was ambulating and tolerating a regular diet. The patient was discharged home with well-healing incisions and no bleeding.

However, the histopathology reports revealed an ovarian sex cord-stromal tumor in favor of a granulosa cell tumor. Microscopic findings included small bland cuboidal to polygonal cells with scant cytoplasm and pale, uniform, angulated, focally grooved nuclei (coffee bean) arranged in various patterns, including diffuse, trabecular, and corded, along with microfollicular forming Call-Exer bodies and few macrofollicules. There was minimal stromal component and few plumbed cells. These cells were positive for inhibin, calretinin, and PANCK, while negative for synaptophysin, HMB45, CK7, EMA, and CD10.

## Discussion

Copper T is a T-shaped device wrapped in a copper wire that works by altering tubal motility. It has an endometrial inflammatory reaction that prevents implantation along with a spermicidal effect. Though complications are rare with IUD placement, they often present as pain, bleeding, migration/displacement outside the uterine cavity, uterine perforation, and pregnancy [4].

While migration is a rare complication of IUD insertion, it can further increase the risk of serious complications such as uterine or bowel perforation [2-4]. In our case, migration was noted to the anterior abdominal wall; however, there was no perforation. Aljohani et al. suggested that the presentation of IUD migration often depends on the location and extent of migration [5]. The patient can be asymptomatic or develop some abdominal pain which might also present with shock-like symptoms in adverse cases [5]. Migration is often noted to the omentum, urinary bladder, cecum, and pouch of Douglas. The mechanism is often not understood but might likely be due to cesarean section or via fallopian tubes or ovary. Akhtar et al. emphasized that migration can occur due to uterine and bladder contraction and peristaltic movements [4]. In our case, we expect the IUD to be displaced during the cesarean section through the fallopian tubes to the anterior abdominal wall which formed a mass attached to the anterior abdominal wall. This was in line with the case reported by Arif and Mohammed [3]. They reported a migrated IUD presenting as an anterior abdominal wall abscess [3].

Often, the displaced IUD cannot be identified by physical examinations and requires radiological investigations [6-9]. Many studies have reported the usage of ultrasonography as the first modality in detecting migrated IUDs. Other studies such as an abdominal X-ray, CT scan, and MRI scan might also be used to diagnose the exact location of the migrated IUD [2,3,5,7,9]. However, it can be missed in radiological investigations and is often misdiagnosed as a hernia, mass, or abscess. In these cases, diagnostic laparoscopy/laparotomy is used to identify the IUDs [4,5,10]. In our case, an MRI scan suggested findings suggestive of a urachal cyst. However, during the laparoscopy, we found the anterior abdominal mass to be an IUD surrounded by a cystic-like lesion.

Migrated IUDs should be retrieved as early as possible to prevent further complications. They can be retrieved by a diagnostic laparoscopy or laparotomy. Minimally invasive techniques could be performed if detected early and can prevent any surgical complications [2,3,6,7]. Hence, it is important to always confirm the presence of an IUD by obtaining a thorough history and radiological investigations. In our case, a laparoscopic adnexal mass excision was performed, and the patient recovered without any complications.

## Conclusions

IUD migration is a rare complication with an incidence of about one per every 1,000 cases reported. Imaging of different types is considered the best option for diagnosis; however, it can be misleading. In cases of high clinical suspicion, other options including laparoscopy should be considered to retrieve the migrated IUDs and prevent long-term complications.
